# PReS-FINAL-2302: Evaluation of magnetic resonance imaging abnormalities in juvenile neuro-psychiatric systemic lupus erythematosus

**DOI:** 10.1186/1546-0096-11-S2-P292

**Published:** 2013-12-05

**Authors:** MRA Al-Obaidi, S Brown, L Ramsden, N Martin, D Saunders, C Pilkington, P Brogan, D Eleftheriou

**Affiliations:** 1 Paediatric and Adolescent Rheumatology, Sheffield Children's NHS Foundation Trust, Sheffield, UK; 2 Paediatric Rheumatology, Great Ormond Street Hospital, London, UK; 3 Paediatric Neuro-Radiology, Great Ormond Street Hospital, London, UK; 4 General Paediatrics, Sheffield Children's NHS Foundation Trust, Sheffield, UK

## Introduction

Neuropsychiatric systemic lupus erythematosus (NPSLE) is a diagnostically challenging, severe, and life-threatening condition. Neuroimaging techniques such as magnetic resonance imaging (MRI) are increasingly used to assist the diagnosis and monitoring of the disease course in adults with NPSLE. The role of MRI in the evaluation of children and adolescents with suspected juvenile NPSLE is however unknown.

## Objectives

The aim of this study was to describe the abnormalities identified with conventional MRI in children with NPSLE; and any potential associations with clinical and/or serological markers presenting to a tertiary paediatric rheumatology service.

## Methods

Single centre (Great Ormond Street Hospital NHS Foundation Trust, London) retrospective case series of patients with juvenile NPSLE seen between April 2003 and October 2010. Contrast enhanced brain MR images of the first episode of active NPSLE were reviewed. All patients fulfilled the American College of Rheumatology (ACR) 1982 revised criteria for the classification of SLE and were classified according to the 1999 ACR case definition for NPSLE syndromes. We excluded patients with a history of alternative neurological conditions. Presenting neuropsychiatric manifestations, immunological findings and treatment are reported. Continuous variables are summarised as median and ranges. Categorical variables are presented as percentages. Fisher's exact test was used to identify the probability of abnormal MRI findings.

## Results

A total of 27 patients median age 11 (4-15) years, 22 females with suspected juvenile NPSLE were studied. Presenting clinical symptoms included: headaches (85.1%); mood disorder/depression (62.9%); seizures (22.2%); acute psychosis (18.5%); cognitive dysfunction (14.8%); movement disorder (14.8%); acute confusional state (14.8%); aseptic meningitis (7.4%); demyelinating syndrome (3.7%); myelopathy (3.7%); dysautonomia (3.7%); and cranial neuropathy (3.7%). The principal MR findings were: 1) absence of MRI abnormalities despite signs and symptoms of active NPSLE (59% of all patients); 2) focal hyperintensities on T2-weighted imaging in both white and grey matter (33%); 3) diffuse cortical grey matter lesions (3.7%); and 4) diffuse brain atrophy (18.5%). The presence of>2 neuropsychiatric manifestations strongly associated with abnormal MRI findings (p = 0.014). Positive dsDNA antibodies were noted in 74% of patients; positive extra nuclear antibodies in 74%, lupus anticoagulant in 15% and anticardiolipin antibodies in 37%. All children were treated with hydroxychloroquine and high doses of intravenous corticosteroids while other treatments used were: intravenous cyclophosphamide (51.8%); rituximab (40.7%); and plasma exchange (11.1%).

## Conclusion

In the present study, no conventional MRI abnormalities were observed in the majority of patients with clinically active NPSLE. The presence of >2 neuropsychiatric manifestations was strongly associated with abnormal MRI. Improved MR techniques and the advent of other alternative diagnostic imaging modalities may improve the detection rate of brain involvement in juvenile NPSLE.

## Disclosure of interest

None declared.

